# Abnormal Photic Entrainment to Phase-Delaying Stimuli in the R6/2 Mouse Model of Huntington's Disease, despite Retinal Responsiveness to Light

**DOI:** 10.1523/ENEURO.0088-19.2019

**Published:** 2019-12-10

**Authors:** Koliane Ouk, Juliet Aungier, Michelle Ware, A. Jennifer Morton

**Affiliations:** Department of Physiology, Development and Neuroscience, University of Cambridge, Cambridge CB2 3DY, United Kingdom

**Keywords:** Huntington's disease, jetlag, light therapy, phase response curve, phase shift, photic entrainment

## Abstract

The circadian clock located in the suprachiasmatic nucleus (SCN) in mammals entrains to ambient light via the retinal photoreceptors. This allows behavioral rhythms to change in synchrony with seasonal and daily changes in light period. Circadian rhythmicity is progressively disrupted in Huntington’s disease (HD) and in HD mouse models such as the transgenic R6/2 line. Although retinal afferent inputs to the SCN are disrupted in R6/2 mice at late stages, they can respond to changes in light/dark cycles, as seen in jet lag and 23 h/d paradigms. To investigate photic entrainment and SCN function in R6/2 mice at different stages of disease, we first assessed the effect on locomotor activity of exposure to a 15 min light pulse given at different times of the day. We then placed the mice under five non-standard light conditions. These were light cycle regimes (T-cycles) of T21 (10.5 h light/dark), T22 (11 h light/dark), T26 (13 h light/dark), constant light, or constant dark. We found a progressive impairment in photic synchronization in R6/2 mice when the stimuli required the SCN to lengthen rhythms (phase-delaying light pulse, T26, or constant light), but normal synchronization to stimuli that required the SCN to shorten rhythms (phase-advancing light pulse and T22). Despite the behavioral abnormalities, we found that *Per1* and c-*fos* gene expression remained photo-inducible in SCN of R6/2 mice. Both the endogenous drift of the R6/2 mouse SCN to shorter periods and its inability to adapt to phase-delaying changes will contribute to the HD circadian dysfunction.

## Abstract (precis)

The circadian clock located in the suprachiasmatic nucleus (SCN) in mammals entrains to ambient light via the retinal photoreceptors. This allows behavioral rhythms to change in synchrony with seasonal and daily changes in light period. Circadian rhythmicity is progressively disrupted in Huntington’s disease (HD) and in HD mouse models such as the transgenic R6/2 line.

## Significance Statement

Circadian dysfunction manifests early in Huntington’s disease (HD). Given the importance of circadian function to human behavior, such deficits have the potential to exacerbate symptoms of HD, particularly cognitive impairment and irritability. Here we tested the ability of a mouse model of HD that exhibits progressive circadian abnormalities (R6/2 line) to adjust their circadian rhythms to environmental cues (photic synchronization). We found that photic synchronization was impaired when the stimuli required the suprachiasmatic nucleus (SCN) to lengthen its rhythms, but normal when the SCN was required to shorten them. Interestingly, light-inducible gene expression still occurred in SCN of R6/2 mice that were behaviorally unresponsive to a light pulse, suggesting that afferent inputs to the SCN were functional.

## Introduction

The circadian clock, located in the suprachiasmatic nucleus (SCN) of the anterior hypothalamus, generates endogenous rhythms that can be reset by ambient light. This is to ensure that our internal body functions are appropriately synchronized and adapted to the light/dark (LD) cycle of the environment. The process of photic entrainment is initiated by retinal photoreceptors that receive, integrate, and transfer the light information via the retinohypothalamic tract to the SCN (Perez-Leon et al., 2006). Circadian timing involves the tight regulation of clock gene expression in the SCN: the dimerization of the positive elements CLOCK and BMAL1 promote the expression of negative elements Period (*Per*) and Cryptochrome (*Cry*). In turn, PER and CRY inhibit the CLOCK/BMAL1 complex, closing the feedback transcriptional loop (for review, see Albrecht, 2006; Ko and Takahashi, 2006). Photic stimulation acutely induces the expression of *Per* genes in the SCN (Albrecht et al., 1997; Shearman et al., 1997; Bae et al., 2001), suggesting their involvement in light-induced circadian shifts.

Huntington’s disease (HD) is a neurodegenerative disease caused by a pathologic CAG repeat expansion in the huntingtin gene. In addition to a complex set of progressive motor, cognitive, and psychiatric symptoms (Bates et al., 2015; Schobel et al., 2017), HD is characterized by a progressive disruption in sleep and circadian rhythms (Aziz et al., 2010; Morton, 2013; van Wamelen et al., 2015). The circadian disruption is recapitulated in multiple mouse models of HD (Morton et al., 2005; Kudo et al., 2011; Lin et al., 2019), including the R6/2 mouse used in this study. Although the circadian disruption observed in R6/2 mice is accompanied at a molecular level by a dysregulation of the clock genes expression in the SCN (Morton et al., 2005), the molecular machinery in the SCN remains functionally intact (Pallier et al., 2007). This suggests that the circadian phenotype is due to dysfunctional circuitry in the R6/2 mice rather than disruption to the molecular clock. The circadian system can be divided into the three components (Brown and Schibler, 1999): the retina and retinal afferents to the SCN that modulate rhythms so they are adapted to the environment; the master clock that generates the rhythms; and the efferents from the SCN that allow the rhythmic information to be spread throughout the body (Cermakian et al., 2001). The first component of the circadian system to be disrupted in HD may be the retinal dysfunction and degeneration that has been described in R6/2 and other HD mice models (Helmlinger et al., 2002; Petrasch-Parwez et al., 2004; Batcha et al., 2012; Ragauskas et al., 2014). A recent study has found deficits in retina function of the R6/2 mouse that might cause disruption of light transmission to the SCN (Ouk et al., 2016b). That study reported a decrease in pupillary light responses (PLRs; or the ability of the pupil to constrict in response to light, a marker of light reception in the retina) that is correlated with downregulation of the photopigments melanopsin and cone opsin in both R6/2 mice and a full-length knock-in mouse model of HD (Ouk et al., 2016b). Behaviorally, however, the situation is complex. The period length of R6/2 mice under a 12 h LD cycle is pathologically shortened (to ∼23 h) as the disease progresses (Wood et al., 2013; Ouk et al., 2017), which is consistent with a progressive insensitivity to light. Nevertheless, symptomatic R6/2 mice remain behaviorally responsive to paradigms involving light manipulations. Bright-light therapy delays circadian rhythm disruption (Cuesta et al., 2014), and R6/2 mice can entrain to a 23 h day and adapt to phase advances in the jet lag paradigm (Wood et al., 2013). Furthermore, variations of photoperiod lengths are able to reverse, accelerate, or delay the circadian disruption (Ouk et al., 2017).

Light is the most powerful cue for resetting the circadian clock in response to photoperiod changes. The phase response curve (PRC) is a particularly useful experimental tool for investigating the responsiveness of an organism to acute light (Daan and Pittendrigh, 1976; Johnson, 1999). In this study, we investigated the mechanisms of light entrainment in R6/2 mice using PRCs to light at different stages of the disease. These were presymptomatic (9 weeks, when mice do not exhibit any HD-related phenotype), early symptomatic stage (12 weeks, when mice show mild HD phenotype), and late symptomatic stage (14 weeks, when mice show severe phenotype). We subsequently assessed the expression of the photo-inducible clock gene *Per1* and the marker of neuronal activity c-*fos* in the SCN of R6/2 mice subjected to light pulses to investigate the molecular mechanisms of photic entrainment. Finally, we tested the limitation of SCN entrainment by placing R6/2 mice under variable length of T-cycles (21, 22, and 26 h) and under conditions of constant light (LL) or constant darkness (DD).

## Materials and Methods

### Animals and housing conditions

All experimental procedures in this study were performed under the Animals (Scientific Procedures) Act 1986 Amendment Regulations 2012 and in accordance with the ethical review by the University of Cambridge Animal Welfare and Ethical Review Body.

Wild-type (WT) and R6/2 mice on a CBA × C57BL/6J background were used. The genotype and determination of the number of CAG repeats was conducted on tail snips by Laragen using GeneMapper and verified after completion of the experiments. In total, 267 R6/2 and 155 WT mice were used in the study. R6/2 mice had a mean (±SEM) CAG repeat of 252 ± 3.

Before the experiments, mice were group-housed in cages of the same sex and genotype. For the measurement of daily activity patterns, mice were individually housed in a ventilated, light-tight and sound-proof Scantainer cabinet (Scanbur) with a built-in light system (minimum, 100 lux). Humidity was maintained at 55 ± 10% and temperature at 22 ± 1°C. Mice had *ad libitum* access to food and water, which was delivered by lowered bottles with elongated spouts to facilitate access for symptomatic R6/2 mice.

Four experiments were conducted (for details, see below). For the entrainment to light pulses experiment, a total of 83 WT and 197 R6/2 mice were used (Table 1, Light pulse experiment). For *in situ* hybridization analysis, 12 WT and R6/2 female mice were exposed to a light pulse for 15 min at the age of 14 weeks and left undisturbed for 45 min before being killed. We only used females for the *in situ* hybridization experiment because of mouse availability (R6/2 males were used for breeding). Since all mice, regardless of sex, responded the same way to light pulse with appropriate advance or delayed phase shift for the corresponding CTs, we expected that the response would be similar in male mice, although we did not measure it. A small group of three WT and three R6/2 mice were chosen at random from this larger set of mice for the *in situ* hybridization experiment after verifying that the light pulse was given at the right circadian time point. For the entrainment to the T-cycles experiment, a total of 55 WT and 53 R6/2 mice were used (Table 1, T-cycle experiment). For the free-running rhythms experiment, a total of 17 WT and 17 R6/2 mice were used (Table 1, Free-running rhythms experiment).

**Table 1:** Numbers and sex of mice used in the light pulse, T-cycle, and free-running rhythm experiments

	WT mice		R6/2					
			9 weeks		12 weeks		14 weeks	
	Males	Females	Males	Females	Males	Females	Males	Females
Light pulse experiment								
Circadian time								
CT0	4	4	7	7	7	8	5	3
CT3	9	6	3	5	6	5	4	3
CT6	7	6	7	6	5	5	5	3
CT9	4	9	5	10	5	7	1	4
CT12	6	8	11	4	6	5	4	8
CT15	4	8	6	4	7	7	4	3
CT18	6	13	8	9	5	6	5	5
CT21	6	4	3	5	3	5	5	5
CT23	6	4	6	6	7	5	5	4
CT24	4	4	7	7	7	8	5	3

	WT		R6/2					
	Males	Females	Males	Females				
T-cycle experiment								
T-cycle								
T21	10	9	8	9				
T22	9	9	9	9				
T26	9	9	9	9				

	WT		R6/2					
	Males	Females	Males	Females				
Free-running rhythms experiment								
T-cycle								
LL and DD	8	8	9	9				

For entrainment to light pulses, mice were used once or twice and were exposed to a single light pulse [Table 1 shows a summary of the number and sex of animals tested at each circadian time (CT)]. At 9 weeks, 112 naïve R6/2 mice were used. At 12 weeks of age, 99 of these mice and 5 naïve mice were subjected to a second light pulse. (Of the original 112 mice, 13 were excluded because of arrhythmicity.) Finally, 80 R6/2 naïve mice were tested at 14 weeks of age. Eighty-three WT mice were used in this experiment. All WT mice were subjected to light pulses at 9 weeks of age, and 30 of those were subjected to a second light pulse between 14 and 30 weeks of age. Since WT mice do not exhibit any photic disruption between 9 and 30 weeks, all data from the WT mice, regardless of the age at which they received the light pulse, were pooled together for analysis and comparison with R6/2 groups. Where mice were tested twice, the light pulses were given at different CTs, with an interval of at least 4 weeks between the light pulses. Mice were excluded from the study if their circadian rhythms became arrhythmic.

### Survival

Survival was measured when a mouse was killed for humane reasons because of their disease, if they became moribund, or if they failed to rouse after gentle stimulation. No WT mice were killed due to ill health during the study.

### Circadian analysis

Circadian activity data were collected from each mouse using passive infrared motion sensors (catalog #DS936, Bosch) placed on top of the cage and connected to a computer with a recording system (ClockLab, Actimetrics). Activity patterns were double plotted in 5 min bins and periodograms were generated with ClockLab software (R2015a, Actimetrics) to analyze circadian parameters of the behavioral rhythms. Period length of circadian rhythms (τ, time to complete one cycle) was calculated with the least-squares regression line fitted to the activity onsets of the data period to analyze, and verified with the χ^2^ periodograms (level of significance set at *p* < 0.001). The duration of active period (α) was determined as the difference between least-squares regression lines fitted respectively to the activity onsets and offsets of the data period to be analyzed. General activity during active and rest periods was determined using the counts provided by the profile activity function of the ClockLab software. The rest/activity ratio was calculated as the amount of activity occurring during the rest period, as a fraction of the amount of activity occurring during the total activity. Phase shifts after a light pulse were calculated automatically after determination of the least-squares fits to the activity onsets for data of the week before the light pulse (t1) and the week after the light pulse (t2). The resulting phase shift was calculated as equal to (t1 − t2) * 24/τ.

### Lighting conditions

**Figure 1.**
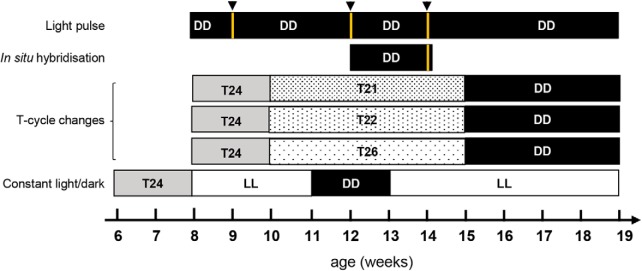
Experimental outline and light schedule for testing. The diagram shows the different light/dark settings for the light pulse, *in situ* hybridization, T-cycle changes (with T24 corresponding to 12 LD cycle; T21 to 10.5 LD cycle; T22 to 11 LD cycle and T26 to 13 LD cycle) and constant light (LL)/dark (DD) experiments. Black arrowheads and yellow lines show the age at which the 15 min light pulse was administered.

The mice were exposed to different light/dark conditions for each experiment. A diagram illustrating the experimental design is shown in Figure 1.

#### Entrainment to light pulses

All mice were singly housed in the circadian cabinet for 7–10 d of habituation under a 12 h LD cycle (lights on at 07:00 A.M. and off at 7:00 P.M.) before the start of the experiment, followed by at least 10–14 d in total darkness (DD) to allow endogenous rhythm expression. Activity was recorded throughout the experimental period. Once the behavioral circadian rhythms of mice were “free running,” the mice received a 15 min light pulse (white light, 300 lux) at different CTs: 0, 3, 6, 9, 12, 15, 18, 21, or 23. After exposure to a single light pulse, mice were placed back in the circadian cabinet under DD to record activity for at least 10–14 d to allow calculation of shifts in activity onsets induced by the light pulse. Any mice receiving a light pulse that became arrhythmic during the study were excluded from the analysis, since the phase shift could not be calculated.

#### Entrainment to T-cycles

Mice were placed at 9 weeks of age in circadian cabinets under a 12 h LD cycle (T24) for 1 week. They were then placed under a T-cycle with a length of T21 (10.5 h LD cycle), T22 (11 h LD cycle), or T26 (13 h LD cycle) for 5 weeks between the ages of 10 and 15 weeks. Then, the mice were placed in DD from 15 weeks of age for the remainder of the experiment.

#### Measuring free-running rhythms under constant light or constant darkness

WT and R6/2 mice were placed in a circadian cabinet from 6 weeks of age under a 12 h LD cycle. Between 8 and 11 weeks of age, mice were exposed to LL. Between 12 and 14 weeks of age, they were then placed under DD. Finally, at 15 weeks of age, they were placed again under LL where they remained until they were 17 weeks of age.

### *In situ* hybridization

Twelve-week-old WT and R6/2 mice were placed in DD for 10–14 d. On day 15, mice were subjected to a 15 min light pulse (white light, 300 lux) at CT6, CT15, or CT23 and placed back in the circadian cabinet in the dark for 45 min. Mice were subsequently killed by cervical dislocation, their brains were collected and immediately snap frozen and stored at −80°C.

Sense and antisense c-*fos* and vasoactive intestinal peptide gene (*vip*) mRNA riboprobes were made by PCR amplification using primers designed from a National Center for Biotechnology Information reference sequence (NM_010234.2 and NM_001313969.1, respectively) and subcloned into the Invitrogen pCRII-TOPO vector (Thermo Fisher Scientific). Sense and antisense *Per1* mRNA riboprobes were obtained from a plasmid received as a gift from Prof. Michihiro Mieda (Mieda et al., 2015) from the Department of Molecular Neuroscience and Integrative Physiology, Faculty of Medicine, Kanazawa University, Japan. An RNA sense probe for each respective gene, c-*fos*, *Per1*, and *Vip*, were used as a negative control for the *in situ* hybridization.

Brain sections were cut coronally at a thickness of 18 μm. Sections were cut in a way that resulted in multiple sections of the SCN on different slides, and we were therefore able to repeat the RNA detection in the same animal and reproduce/confirm the results. Slides were washed with 1× PBS with 0.1% Tween 20, permeabilized with proteinase K, followed by acetylation solution (0.05% acetic anhydride in 0.1 m triethanolamine). For hybridization, digoxigenin-labeled probes (1:100) were added to prewarmed prehybridization mix (50% formamide, 6× SSC, 5× Denhardt’s solution, 0.5% SDS, 100 μg/ml tRNA) at 70°C overnight. To wash the unbound probe, slides were washed with 5× SSC followed by 0.2× SSC. The slides were washed with 1× maleic acid buffer containing 0.1% Tween 20. The slides were blocked with blocking solution (2% Boehringer blocking reagent and 20% goat serum in 1× maleic acid buffer containing 0.1% Tween 20) for 1 h. Alkaline phosphatase-conjugated anti-digoxigenin antibody (catalog #AB_2734716, Roche) was added to the blocking solution at 1:2000 and incubated at 4°C overnight. The slides were washed with 1× maleic acid buffer containing 0.1% Tween 20, followed by alkaline phosphatase buffer (NTMT: 100 mm NaCl, 100 mm Tris-HCl, pH9.5, 50 mm MgCl_2_, 1% Tween 20). The substrate (13 μl of nitro blue tetrazolium and 10.5 μl of 5-Bromo-4-chloro-3-indolyl phosphate) was added to NTMT. Once the color had developed to the desired intensity, which was predetermined and constant for all slides at ∼48 h, the slides were washed in 1× PBS. Images were taken using a Nikon Eclipse 80i microscope with an MBF Bioscience camera and Stereo Investigator 10.54 software (MBF Bioscience; RRID:SCR_002526) and have not been analyzed or manipulated further. Since *Per1* and c-*fos* RNA expressions are induced by light pulse, we were therefore interested in the qualitative detection of the RNA. As the increase (or not) of RNA expression after light pulse was obvious in the images, we did not attempt to quantify the expression levels.

### Statistics

All data are expressed as the mean ± SEM. Statistical differences were analyzed using StatSoft Statistica 19.0 software (version 12, StatSoft) or Prism (version 7.00, GraphPad Software). When applicable, normality distribution was tested with the D’Agostino and Pearson normality test. All data were initially analyzed for each sex separately. However, the goal of our experiments was not to study sex dimorphism, but to study the effect of HD mutation in disrupting photic synchronization. For the light pulse experiment, phase shifts in response to the light pulse were in the same direction for both sexes (i.e., both either increased or decreased depending on the time of the light pulse), and no sex differences were found. Therefore, for each experiment (light pulses, T-cycle, and LL or DD), data from both sexes were pooled for clarity of presentation. For the light pulse experiment, differences between groups were analyzed with a Kruskal–Wallis test followed by a Dunn’s multiple-comparisons test. For the T-cycles, LL, and DD experiments, differences between groups were analyzed using an ANOVA with repeated measures, followed by Bonferroni *post hoc* test. The level of significance was set at *p* < 0.05. All statistical tests are presented in Table 2.

**Table 2:** Statistical table

	Comparison	Data structure	Type of test	Power
a	PRC CT18 WT vs 9 week R6/2	Normal distribution	Kruskal–Wallis test, *post hoc* Dunn's multiple-comparisons test	*p* = 0.0375
b	PRC CT3 WT vs 9 week R6/2	Normal distribution for WT, *N* too small for 9-week-old R6/2	Kruskal–Wallis test, *post hoc* Dunn's multiple-comparisons test	*p* = 0.0002
c	PRC CT23 WT vs 9 week R6/2	Normal distribution	Kruskal–Wallis test, *post hoc* Dunn's multiple-comparisons test	*p* = 0.0068
d	PRC CT24 WT vs 9 week R6/2	Normal distribution for R6/2, not for WT	Kruskal–Wallis test, *post hoc* Dunn's multiple-comparisons test	*p* = 0.0196
e	PRC CT15 WT vs 12 week R6/2	Normal distribution	Kruskal–Wallis test, *post hoc* Dunn's multiple-comparisons test	*p* = 0.0010
f	PRC CT18 WT vs 12 week R6/2	Normal distribution	Kruskal–Wallis test, *post hoc* Dunn's multiple-comparisons test	*p* = 0.0097
g	PRC CT24 9 vs 12 week R6/2	Normal distribution for R6/2, not for WT	Kruskal–Wallis test, *post hoc* Dunn's multiple-comparisons test	n.s., *p* = 0.0789
h	PRC CT15 12 vs 14 week R6/2	Normal distribution	Kruskal–Wallis test, *post hoc* Dunn's multiple-comparisons test	n.s., *p* = 0.5049
i	PRC CT18 12 vs 14 week R6/2	Normal distribution	Kruskal–Wallis test, *post hoc* Dunn's multiple-comparisons test	n.s., *p* > 0.9999
j, k, l	τ under T26 WT & R6/2	Normal distribution for R6/2, not for WT	Repeated-measures ANOVA, *post hoc* Bonferroni test	*p* = 8.38E-8; *p* = 4E-5; *p* = 9.15E-10
m	α under T26 WT & R6/2	Normal distribution for R6/2, not for WT	Repeated-measures ANOVA, *post hoc* Bonferroni test	*p* = 2.43E-06
n	Survival curves under T21 vs T22	-	Log-rank (Mantel–Cox) test	*p* = 0.0035
0	Survival curves under T21 vs T24	-	Log-rank (Mantel–Cox) test	*p* = 0.0025
p	Survival curves under T21 vs T26	-	Log-rank (Mantel–Cox) test	*p* = 0.0050
q, r, s	τ under constant light R6/2 vs WT	Normal distribution	Repeated-measures ANOVA, *post hoc* Bonferroni test	*p* = 0.016; *p* = 5.42E-10; *p* = 0.017
t, u	α under constant light R6/2 vs WT	Normal distribution	Repeated-measures ANOVA, *post hoc* Bonferroni test	*p* = 8.49E-6; *p* = 2.47E-13
v	τ under second constant light R6/2 vs WT	Normal distribution	Repeated-measures ANOVA, *post hoc* Bonferroni test	*p* = 5.22E-07
w	Period length under DD WT vs R6/2	Normal distribution	Repeated-measures ANOVA, *post hoc* Bonferroni test	*p* = 3E-4
x	Amplitude of rhythms under DD WT vs R6/2	Normal distribution	Repeated-measures ANOVA, *post hoc* Bonferroni test	*p* = 0.0006
y	Activity counts under DD WT vs R6/2	Normal distribution for R6/2, not for WT	Repeated-measures ANOVA, *post hoc* Bonferroni test	*p* = 6E-5

The letter for each line of the table refer to the numerical values provided in the text as they appear in the Results.

## Results

### Progressive alteration in response to light pulse in R6/2 mice

**Figure 2.**
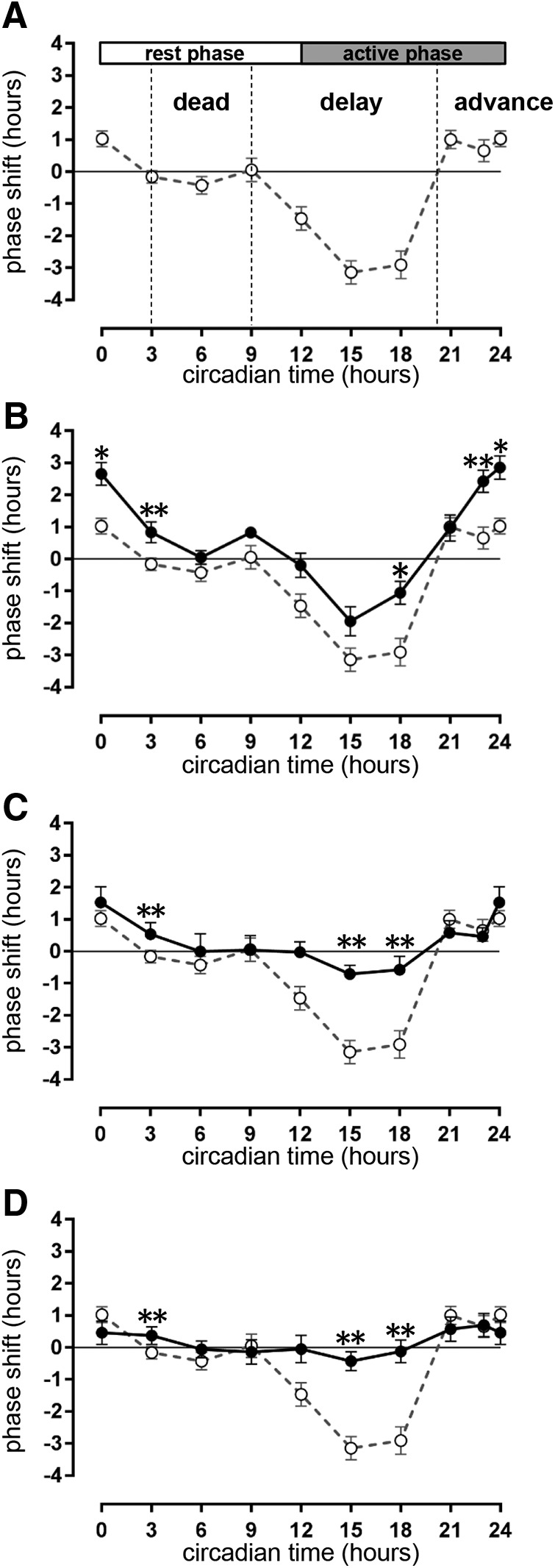
Phase responses to a single light pulse in WT and R6/2 mice. ***A–D***, Graphs show phase response curves in response to a 15 min light pulse at 300 lux for WT mice (open symbols in ***A–D***) and R6/2 mice (solid symbols) at presymptomatic (***B***; 9 weeks of age), early symptomatic (***C***; 12 weeks of age) and symptomatic (***D***; 14 weeks of age) time periods. Data for CTs are double plotted at CT0 and CT24 for clarity of presentation. The WT curve from ***A*** is replicated in ***B–D*** (dashed line) for easy comparison with the R6/2 data (solid lines). Vertical black dash lines indicate the boundaries of the dead zone, delay zone, and advance zone. All data are shown as the mean ± SEM with a minimum of *n* = 3 at each time point (*p < 0.05; **p < 0.01).

**Figure 3.**
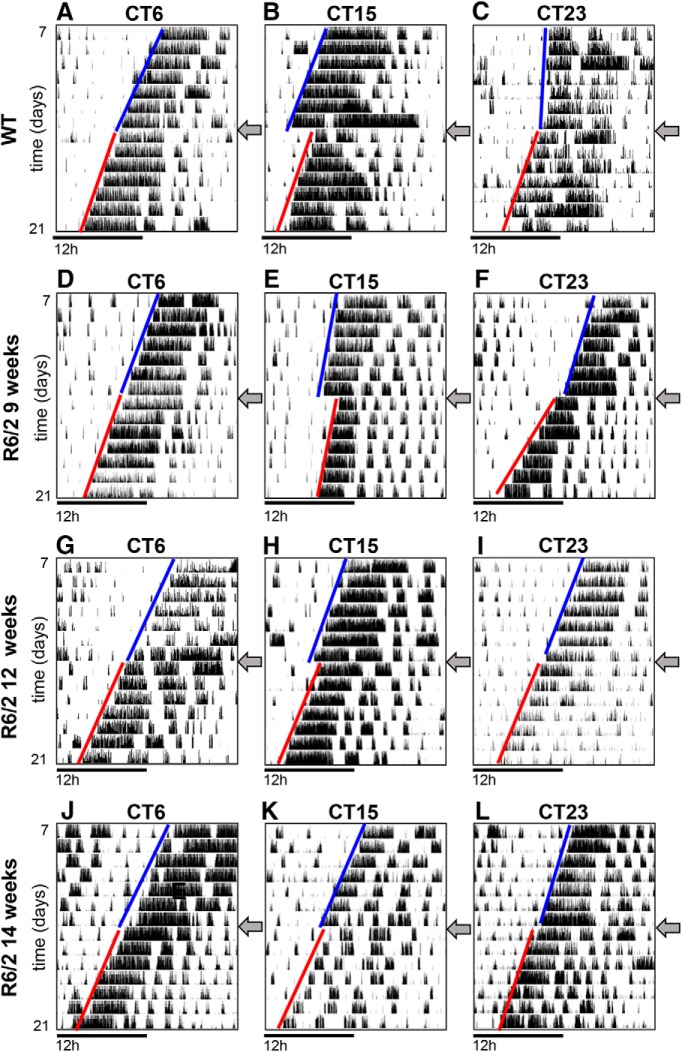
Shifts in activity onset induced by light pulses in R6/2 and WT mice. ***A–L***, Representative single-plotted actograms of WT mice (***A–C***) and R6/2 mice at the age of 9 weeks (***D–F***), 12 weeks (***G–I***), and 14 weeks (***J–L***) placed under constant darkness and subjected to a single 15 min light pulse (300 lux) at CT6, CT15, or CT23. The arrow at the right of each actogram indicates the day when the light pulse was administered. The blue lines indicate the activity onset for 7 d before the light pulse, and the red lines indicate the activity onsets for the 7 d following the light pulse.

After exposing mice to a light pulse at various CTs throughout a 24 h period, we obtained a PRC. The PRC to light of WT mice with a CBA×C57BL/6 background (Fig. 2*A*) exhibited the same characteristics of the PRC obtained from pure C57BL/6 mice reported previously in the literature (Pendergast et al., 2010). Three clearly identifiable regions of the curve were observed, namely a “dead” region during most of the subjective day (CT3–CT9), where the light pulse did not induce any shift in the activity onset on subsequent days; a “phase delay” region during the early subjective night (CT9–CT18) that caused a phase delay of ∼3.2 h at CT15; and a “phase advance” region during late subjective night (CT21–CT24) that caused a phase advance of ∼1 h at CT24 (Figs. 2*A*, 3*A–C*).

The shape of the PRC to light pulse obtained in R6/2 mice at 9 weeks of age (Fig. 2*B*) resembled that of WT mice, with a dead region (no response), a phase delay region, and a phase advance region. However, the magnitude of the shifts was different (Figs. 2*B*, 3*D–F*), with significantly smaller shifts in R6/2 mice in the delay region at CT18 (*p* = 0.04)^a^, and significantly larger shifts at CT3 (*p* < 0.001)^b^ and in the advance region at CT23 (*p* = 0.007)^c^ and CT24 (*p* = 0.02)^d^. With increasing age and consequently disease progression, the PRC progressively flattened. By 12 weeks of age, the phase shifts were significantly smaller only in the delay region at CT15 (*p* = 0.001; Figs. 2*C*, 3*H*)^e^ and CT18 (*p* = 0.01; Fig. 2*C*)^f^, and the differences seen at 9 weeks of age in the advance region were abolished (Figs. 2*C*, 3*I*)^g^. At 14 weeks of age, R6/2 mice subjected to light pulses were still rhythmic (Fig. 3*J–L*), but the resulting phase shifts at CT15^h^ and CT18^i^ were no longer existent (Figs. 2*D*, 3*K*).

**Figure 4.**
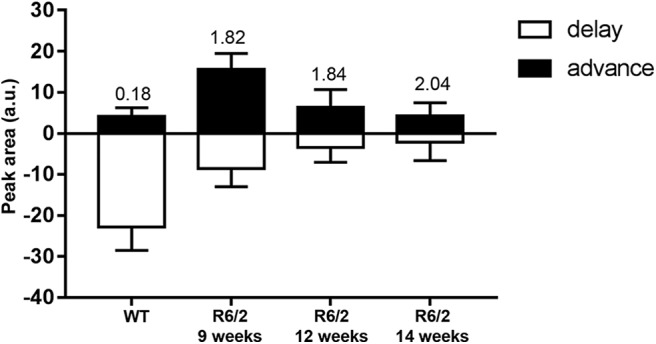
The advance/delay ratio of the phase response curves is altered in R6/2 mice. Graphs show the change of peak area of the phase response curve in response to a 15 min light pulse at 300 lux for R6/2 mice at 9, 12, and 14 weeks of age and for WT mice. The value of the advance/delay ratio is shown above the bars on the graph.

The shape of the WT PRC was asymmetrical, with the delay region larger than the advance region (Fig. 4). The PRCs from R6/2 mice were also asymmetrical regardless of the age, but this time the delay region was smaller than the advance region.

### Normal expression of *Per1* and c-*fos* is induced in the SCN of R6/2 mice after a light pulse

To establish whether the progressive changes in PRC of R6/2 mice were related to the disruption of light-inducible gene expression in the SCN, we conducted *in situ* hybridization to analyze the expression of *Per1* and c-*fos* after a light pulse in three WT and three R6/2 mice at 14 weeks of age. The SCNs of all three mice tested showed the same expression patterns before and after a light pulse for c-*fos*, *Per1*, and *Vip* for all the circadian times tested regardless of genotype.

**Figure 5.**
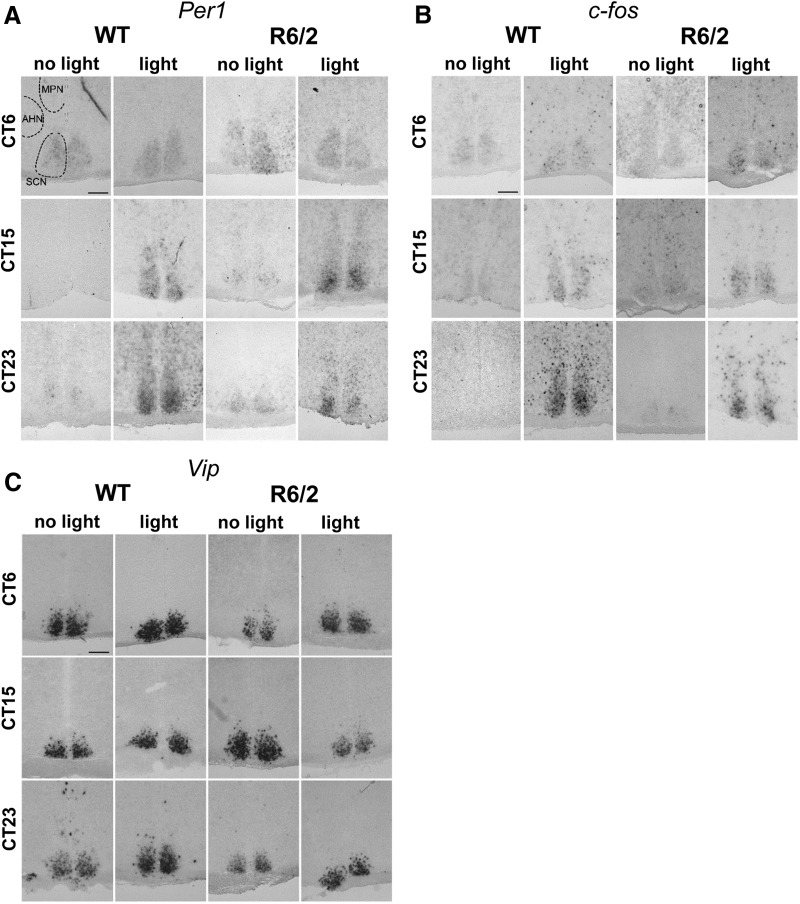
Expression of *Per1* and c-*fos* in the SCN induced by a single light pulse in WT and R6/2 mice. ***A–C***, Representative photomicrographs show mRNA expression in the SCN by *in situ* hybridization of *Per1* (***A***), c-*fos* (***B***), and *Vip* (***C***) in 14-week-old R6/2 and WT mice under DD or 45 min after a light pulse (15 min, 300 lux minimum) at CT6, CT15, or CT23. The SCN, medial preoptic nucleus (MPN), and anterior hypothalamic nucleus (AHN) are indicated with dashed outlines. Scale bar, 200 μm.

Results obtained were similar at all the ages tested (data not shown). Consistent with the literature with normal mice placed under 12 h LD cycle (Shearman et al., 1997, Bae et al., 2001), we found *Per1* expression in control conditions (DD, no light pulse) was high during the rest period at CT6 and low during the active period at CT15 and CT23 in both WT and R6/2 SCN (14-week-old mice are shown in Fig. 5*A*). *Per1* expression was upregulated after a light pulse in both WT and R6/2 mice during the subjective night at CT15 and CT23 (Fig. 5*A*). In WT and R6/2 mice, c-*fos* expression in basal conditions in DD was low for all CTs, and was induced after a light pulse given during the subjective night at CT15 and CT23, but not during the subjective day at CT6 (Fig. 5*B*). This was consistent with the literature for WT mice placed under a 12 h LD cycle (Colwell and Foster, 1992). The levels of expression of *Vip*, an SCN neuropeptide output involved in rhythmicity, were also similar in WT and R6/2 mice, both in basal conditions and after a light pulse (Fig. 5*C*).

### Normal entrainment to short days of 22 h, but abnormal entrainment to long days of 26 h

**Figure 6.**
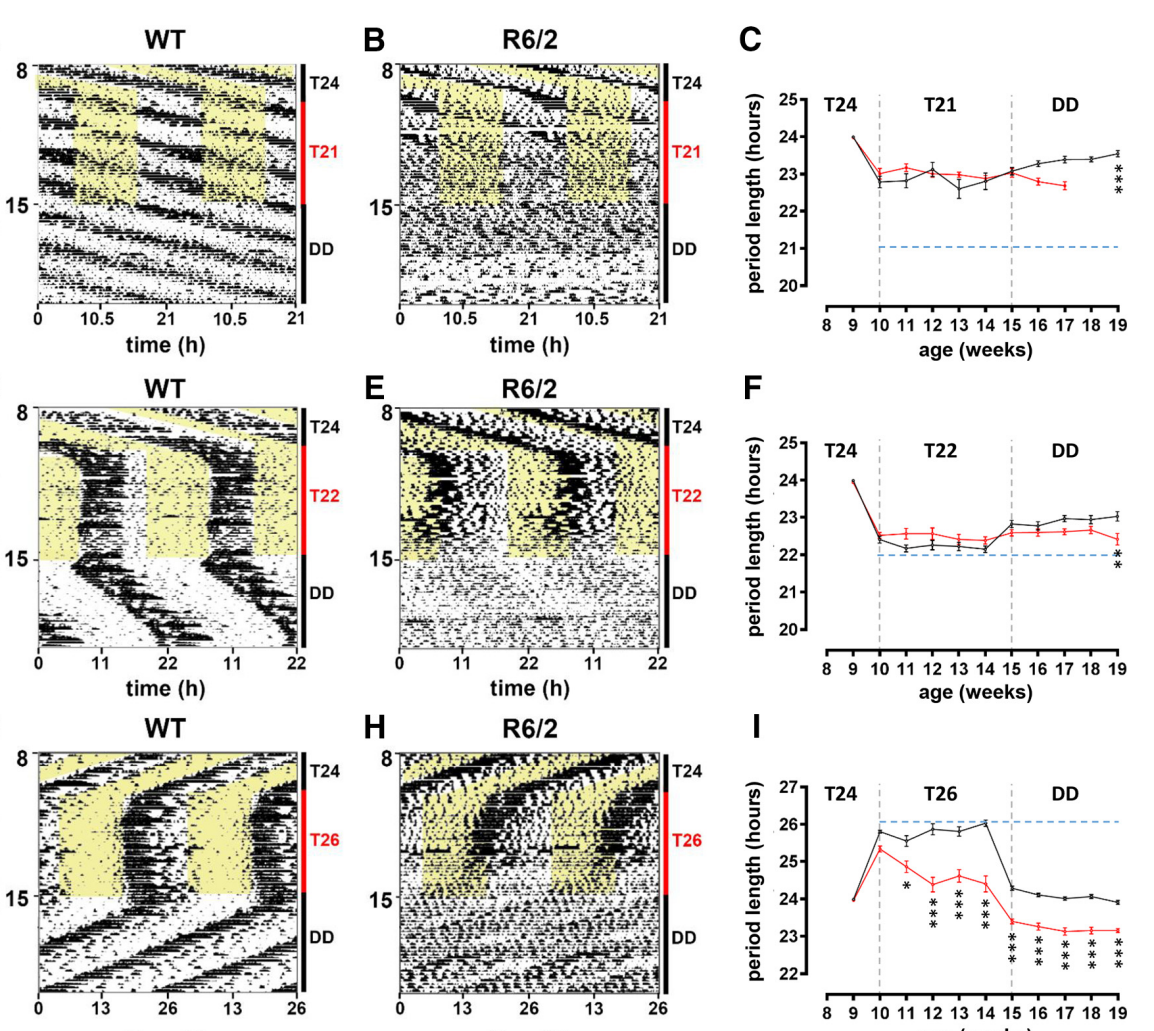
Rest–activity patterns of WT and R6/2 mice under T-cycles of 21, 22, and 26 h. ***A***, ***B***, ***D***, ***E***, ***G***, ***H***, Rest–activity profiles of WT mice (***A***, ***D***, ***G***) and R6/2 mice (***B***, ***E***, ***H***) placed under T24 then T21 (***A***, ***B***), T22 (***D***, ***E***), or T26 (***G***, ***H***), and, finally, constant darkness. ***C***, ***F***, ***I***, Graphs show the evolution of period length during the changes in light schedules. *p < 0.05; **p < 0.01; ***p < 0.001. ***C***, ***F***, ***I***, The blue dotted line shows the time period of the light settings, or where the light had been set previously for the part of the plot where the mice were in DD.

To confirm the disrupted circadian phenotype seen with the PRCs (e.g., an altered delayed phase shift in symptomatic R6/2 mice), we investigated the stability range of entrainment of R6/2 mice by analyzing their entrainment to short or long LD T-cycles. Under the T21 cycle, the WT and R6/2 mice did not entrain (Fig. 6*A*,*B*). Only 1 of 19 WT mice synchronized to T21. Both WT and R6/2 mice exhibited significant negative masking (light-mediated suppression of locomotor activity in rest phase; Mrosovsky, 1999). All mice showed clear endogenous rhythms for the 5 weeks they were under the T21 cycle, albeit with mean period lengths >21 h (WT mice, 22.8 ± 0.02 h; R6/2 mice, 22.8 ± 0.02 h; Fig. 6*C*). Under T22, both WT and R6/2 mice entrained to the LD cycle (Fig. 6*D*,*E*), and the mean period length for the 5 weeks under the T22 cycle was not significantly different between genotypes (WT mice, 22.2 ± 0.02 h; R6/2 mice, 22.5 ± 0.01 h; Fig. 6*F*). The entrainment of R6/2 mice to T22 was, however, not the same as that of the WT mice, which started their locomotor activity exactly at the time of lights off. Rather, R6/2 mice sustained a 22 h rhythm for a few weeks of the T22 application, with a start of their active period that was shifted in the light period. The 22 h rhythm was then disrupted with further shifting of the active period in the light period, leading progressively to rhythm disintegration. Under T26, WT mice entrained to the T-cycle for the whole of the experimental period (Fig. 6*G*). R6/2 mice, however, entrained to T26 only during the first 2 weeks (Fig. 6*H*). After that, they were unable to sustain the long rhythm and their period length gradually shortened (Fig. 6*H*,*I*). There was a main effect of both genotype (*F*_(1,34)_ = 46.04; *p* = 8,38E-8^j^) and age (*F*_(4,136)_ = 7.00; *p* = 4E-5^k^) on period length, as well as interactions between these two factors (*F*_(4,136)_ = 14.29; *p* = 9.15E-10^l^).

**Figure 7.**
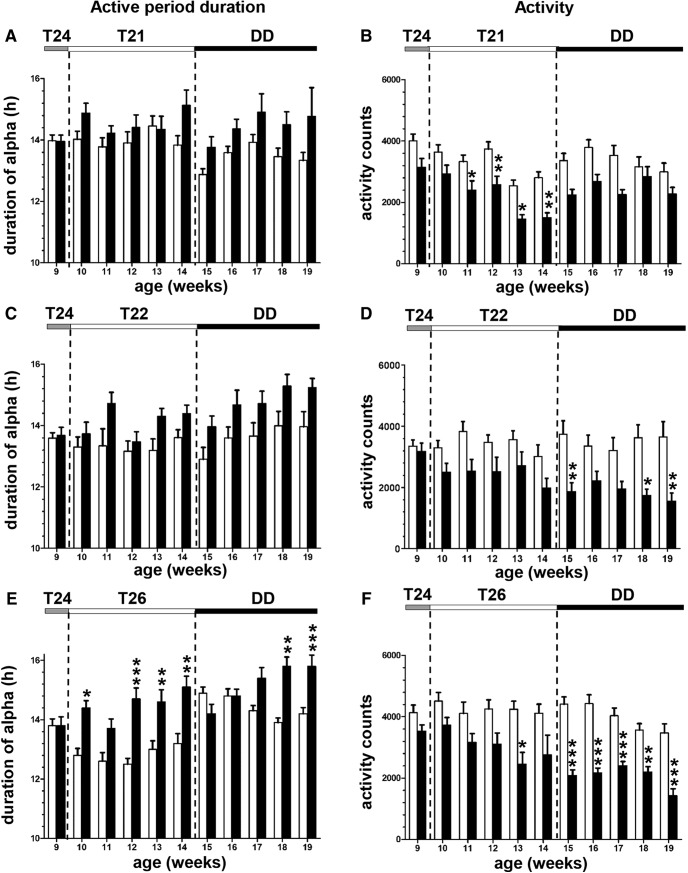
Circadian parameters analyzed in R6/2 and WT mice placed under different T-cycle lengths. ***A–F***, Duration of the active period (***A***, ***C***, ***E***) and total activity counts (***B***, ***D***, ***F***) were analyzed throughout the experiments when WT mice (black histograms) and R6/2 mice (white histograms) were placed under T24 at 9 weeks of age and then under either T21 (***A***, ***B***), T22 (***C***, ***D***), or T26 (***E***, ***F***) for 5 weeks, and, finally, under constant darkness for 5 weeks to analyze after-effects.

There were no significant differences in the duration of the active period between WT and R6/2 mice under T21 or T22 (Figs. 7*A*,*C*), but there was a main effect of genotype under T26 (*F*_(1,34)_ = 31.96; *p* < 0.001^m^; Fig. 7*E*), with a significant increase in the duration of the active period in R6/2 mice compared with WT mice.

The endogenous period following exposure to T26 (Fig. 6*I*), was significantly shorter in all R6/2 mice placed in DD compared with WT mice, but only at the last time point tested for T21 (Fig. 6*C*) and T22 (Fig. 6*F*). In R6/2 mice, significant increases in α were observed only under T26 and activity counts were significantly decreased only under T22 and T26 compared with WT mice (Fig. 7).

**Figure 8.**
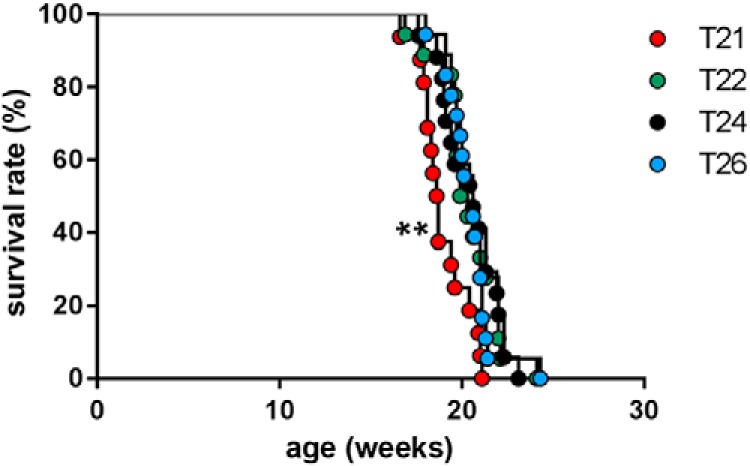
Survival of R6/2 mice exposed to different period lengths. Kaplan***–***Meier survival curves are shown for R6/2 mice placed under T24 (black symbols), T21 (red symbols), T22 (green symbols), and T26 (blue symbols). Survival was significantly different between mice placed under T21 compared with each of the other T-cycles tested (Log-rank Mantel***–***Cox test, *p* < 0.01). No significant difference in survival was seen among T22, T24, and T26. **p < 0.01.

Finally, we found that subjecting the R6/2 mice to T21 was deleterious to their life span (median survival time, 18.7 weeks), which was significantly decreased, compared with R6/2 mice exposed to T22 (median survival time, 19.8 weeks; *p* = 0.0035^n^), T24 (median survival time, 20.8 weeks; *p* = 0.0025^°^), and T26 (median survival time, 20.6 weeks; *p* = 0.005^p^; Fig. 8). There was no difference in survival between R6/2 and WT mice exposed to T22, T24, and T26 cycles.

### Entrainment to constant light and constant darkness in R6/2 mice

**Figure 9.**
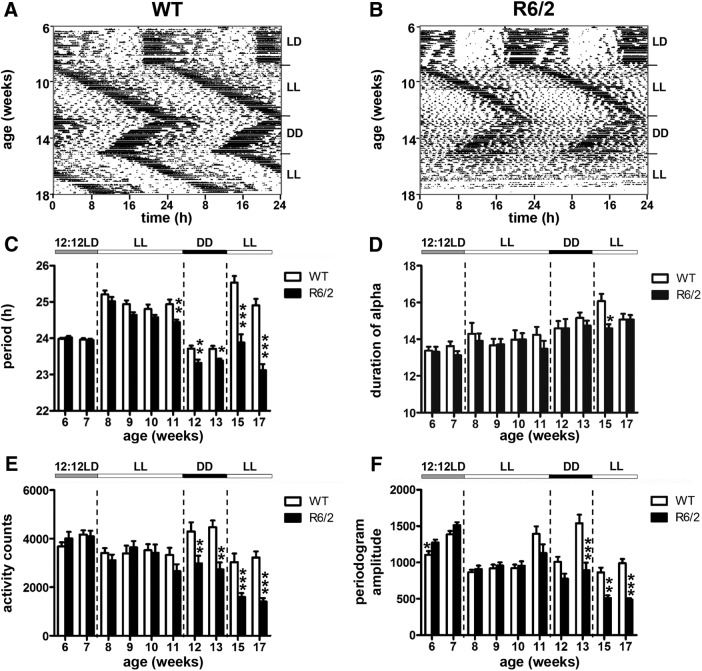
Rest–activity patterns of R6/2 mice under light cycle changes among T24, constant light, and constant darkness. ***A***, ***B***, Rest activity profiles are shown for representative WT mice (***A***) and R6/2 mice (***B***) placed under a succession of light conditions, as follows: a 12 h LD cycle from 6 to 7 weeks of age, LL from 8 to 11 weeks of age, DD from 12 to 14 weeks of age, and, finally, back to LL from 15 to 17 weeks of age. ***C–F***, Histograms show the changes in period length (***C***), the duration of the active period (***D***), general activity (***E***), and the amplitude of the rhythms (***F***) according to the light conditions for WT mice (open columns) and R6/2 mice (filled columns). An asterisk indicates a main effect of genotype: *p < 0.05; **p < 0.01; ***p < 0.001. There was no main effect of time. The bar above the histograms indicates the light condition under which the mice were placed.

To investigate the propensity of the circadian clock in R6/2 mice to accelerate or decelerate rhythms, and to determine whether or not time cues masked a circadian phenotype, we subjected mice to constant conditions (LL cycles interspaced with DD) and measured the free-running circadian periods of the SCN (Fig. 9*A*,*B*). In WT mice, the period length of the free-running rhythms differs slightly from 24 h, with periods typically >24 h under LL and <24 h under DD (Aschoff, 1960). During the first exposure to LL at the presymptomatic stage, R6/2 mice initially responded in a manner similar to WT mice, with an increased period length. However, R6/2 mice were unable to sustain this increase in period length. By 11 weeks of age, there was a main effect on period length of both genotype (*F*_(1,31)_ = 6.4; *p* = 0.016^q^) and time (*F*_(3,93)_ = 19.8; *p* = 5.42E-10^r^), with period length significantly shorter in R6/2 mice than WT mice (*p* = 0.017^s^; Fig. 9*C*). The duration of the active period in R6/2 mice was similar to that in WT mice (Fig. 9*D*), but there was a main effect of age on both activity (*F*_(3,96)_ = 10; *p* = 8.49E-6^t^; Fig. 9*E*) and amplitude of rhythms (*F*_(3,96)_ = 28.70; *p* = 2.47E-13^u^; Fig. 9*F*). When we re-exposed the mice to LL at an older age (between 15 and 17 weeks), the R6/2 mice period length remained shorter than 24 h (23.12 ± 0.17 h at 17 weeks) compared with WT mice that increased (24.91 ± 0.18 h; *p* = 5,22E-07^v^). We found a main effect of genotype on period length, activity levels, and amplitude of rhythms, all of which decreased significantly in R6/2 mice compared with WT mice (Fig. 9*C*,*E*,*F*).

As seen previously under DD, with age R6/2 mice exhibited a progressively shorter period length than was seen in WT mice (Wood et al., 2013; Ouk et al., 2017). In mice under DD at 12–13 weeks of age, there was a main effect of genotype on period length (*F*_(1,32)_ = 16.2; *p* = 3E-4^w^), amplitude of rhythms (*F*_(1,32)_ = 14.41; *p* = 0,0006^x^), and activity counts (*F*_(1,32)_ = 21.43; *p* = 6E-5^y^), with all these parameters significantly decreased in R6/2 mice compared with WT mice (Fig. 9*C*,*E*,*F*).

## Discussion

We show that R6/2 mice are unable to entrain adequately to experimental environmental light changes from ∼11 weeks of age (presymptomatic stage), and that it is more problematic when a deceleration of the period of their circadian rhythms is required than it is when the period requires accelerating. R6/2 mice could neither generate phase delays (when housed under DD and subjected to light pulses), nor adapt and maintain their rhythms to long T-cycles (13 h LD cycle). However, the R6/2 mice were able to accelerate their rhythms, as they could both generate phase advances to a light pulse and adapt to a 22 h T-cycle. The differences we see with WT mice cannot be attributed to aging per se (since the mice are still young) but rather to a possible effect of the HD mutation on aging.

The SCN synchronizes to the LD of the environment through the action of light received by the light-sensitive retinal ganglion cells (Panda et al., 2002; Ruby et al., 2002). Both R6/2 and Q175 mice—a HD model with slower phenotype development—exhibit progressive retinal morphologic changes, including a downregulation in the expression of melanopsin and cone opsin, markers of retinal light-sensitive and cone cells, respectively (Ouk et al., 2016b; Lin et al., 2019). Photodetection appeared to be progressively impaired in R6/2 mice because they exhibited attenuation in their PLR from 12 weeks of age for light at low intensity. Their PLR was disrupted by bright light intensity only at a late stage of disease, suggesting that up to that age light photoreception was still functional, provided the light intensity was sufficiently high. It has been suggested that the photic entrainment requires the integration by the circadian timing system of both the intensity and the duration of photic information (Nelson and Takahashi, 1991; Kornhauser et al., 1996; Morin and Allen, 2006). Therefore, our results support the evidence that there is dysfunction in the retinal inputs to the SCN that means the SCN is unable to reset to the LD cycle of the environment if the light is too dim. This would have consequences in HD patients living in normal lighting levels, since they may exhibit circadian dysfunction. Bright-light therapy may be beneficial in HD mice simply because the light is of sufficient intensity (Cuesta et al., 2014).

Two types of models have been characterized to interpret the entrainment of the circadian timing system to the environmental LD cycle (Pittendrigh and Daan, 1976), as follows: the nonparametric (discrete) and the parametric (continuous) models. The parametric model suggests that the circadian clock continuously adjusts to the light intensity by decelerating and accelerating its rhythms to entrain to the LD cycle. The nonparametric model suggests that discrete light at specific times of day induce entrainment of the circadian clock with phase shifts equal in magnitude to the difference between the period of the rhythm and the period of the LD cycle (Decoursey, 1960; Pittendrigh, 1960). We tested the nonparametric model with the PRC to light and revealed early phenotypes in R6/2 mice whereby they exhibited larger phase advance but smaller phase delay shifts to light pulses than were seen in WT mice. This suggests that the SCN in R6/2 mice is able to accelerate the endogenous rhythms more efficiently but to decelerate them less efficiently than the WT mice. This early phenotype for phase-advanced shifts had almost disappeared by the time the R6/2 mice were tested at 12 and 14 weeks of age, coinciding with the start of a decline in the health of the mice while the phenotype for phase-delayed shifts worsened. Given that symptomatic R6/2 mice develop a dysregulation in *Per2* circadian expression (Morton et al., 2005), our results are consistent with an earlier study that demonstrated that *Per1^−/−^* and *Per2^−/−^* mice had larger phase advances than WT mice (Pendergast et al., 2010). Although we did not measure *Per* gene expression at 9 weeks in the R6/2 250CAG mice, in which the phase advances are larger than those seen in WT mice, we speculate that an early dysregulation in the mRNA expression and protein levels of *Per 1* and/or *Per 2* might be seen at this age.

By 14 weeks, while all the R6/2 mice tested exhibited behavioral rhythmicity, their behavioral responses to a light pulse were almost abolished in the phase delay section of the curve compared with WT mice. Therefore, it seems that the deficiency in the circadian timing system in R6/2 mice is more pronounced at specific circadian phases, with the R6/2 SCN specifically unable to “reset” in response to a light cue administered in the early night.

Using short or long T-cycles, but also constant conditions, we were able to confirm the circadian clock propensity of the R6/2 mice to accelerate rather than decelerate its rhythms.

The period length of R6/2 mice shortens pathologically under both DD and LL. This is also the case under a 12 h LD cycle, when the period length shortens to ∼23 h as the disease progresses (Wood et al., 2013; Ouk et al., 2017). It is, therefore, not surprising that R6/2 mice can entrain more easily to T22 than to T26 since T22 is closer to their endogenous rhythms and required slowing down the molecular machinery of the SCN. Entraining to T26 would require them to adjust by decelerating their endogenous circadian rhythms by ∼3 h each day. Neither WT nor R6/2 mice entrained to T21, suggesting that this exceeded the range of entrainment of their SCN. Interestingly, T21 had a deleterious effect on the survival of R6/2 mice compared with T22, T24, or T26. The cause of this negative impact on the health of R6/2 mice is unknown.

R6/2 mice are arrhythmic at 16 weeks of age, when the behavioral circadian disruption is accompanied by a disruption in clock gene expression (*Per2*, *Bmal1*) in the SCN (Morton et al., 2005; Pallier et al., 2007; Maywood et al., 2010). The molecular clock in the SCN from 16-week-old R6/2 mice is, however, able to function normally *in vitro* (Morton et al., 2005; Pallier et al., 2007; Williams et al., 2011). *Per1* in particular has been shown to be important for photic entrainment in the mammalian circadian system (Pendergast et al., 2010) with *Per1* expression being directly induced by phase-resetting light pulses (Kuhlman et al., 2003; Schwartz et al., 2011). c-*fos* expression in the SCN is also induced by light (Masana et al., 1996). In hamsters, c-*fos* expression was light induced only at phases where phase resetting occurred (Kornhauser et al., 1996). We found that the expression of *Per1* and c-*fos* were light inducible at CT15 and CT23 in 9- and 12-week-old mice that exhibited phase shifts following a light pulse. We found that the SCN of 14-week-old R6/2 mice, which is still rhythmic but does not generate phase delays, is still able to respond with clock gene expression at CT15 and CT23. The disappearance of the behavioral shifts might be explained by a change in the localization of *Per1* expression in the SCN, since an interesting study suggested that the spreading of light-induced *Per* gene expression from the core to the shell of the SCN is necessary for a behavioral phase shift to be produced, with *Per1* expression in the shell associated with phase advances, and *Per2* with phase delays (Yan and Silver, 2002). Our *in situ* hybridization results from symptomatic R6/2 (Fig. 5*A*) mice show a *Per1* expression essentially confined to the core of the SCN. We did not perform *in situ* hybridization in 9-week-old R6/2 mice, and examining the timing of gene expression in the core/shell of the SCN was beyond the scope of this study. It would, however, be interesting to see whether there is a *Per1* light-induced expression in the shell of the SCN at CT21, since that would be consistent with the phase advance we see at that age.

While our *Per1* light-induced expression data support previous findings that the molecular machinery of the SCN is intact, they also indicate that retinal dysfunction is unlikely to be the sole cause of dysrhythmia in R6/2 mice. In support of this suggestion, previous studies have shown that R6/2 mice respond to bright light (Cuesta et al., 2014), jet lag (Wood et al., 2013), and photoperiod length variation (Ouk et al., 2017). This raises the possibility that the involvement of downstream areas of the brain innervated by the SCN causes the disruption of the behavioral response to the phase-delaying light pulse. Unlike the circadian afferents to SCN, circadian efferents from the SCN are not well documented, although several signaling factors in the SCN have been identified as being involved in the circadian regulation of locomotor activity (Li et al., 2012). These include vasopressin, prokineticin 2, TGFα (transforming growth factor α), and cardiotrophin-like cytokine, which are all able to inhibit locomotor activity when they are infused in the third ventricle of mice during the night period. There is currently no evidence for which brain areas are involved in R6/2 mice, but we speculate that candidate regions could include the intergeniculate leaflet, which plays a role in photic entrainment and receives input from both the retina and SCN (Edelstein and Amir, 1999). Hypothalamic regions are also good candidates as alterations have been reported both in HD patients and animal models (Vercruysse et al., 2018; Cheong et al., 2019). The dorsomedial hypothalamic nucleus is responsible for relaying circadian temporal information from the SCN to the locus ceruleus (Gompf and Aston-Jones, 2008), and excitotoxic lesion of this region has been reported to decrease behavioral circadian rhythms including locomotor activity (Chou et al., 2003). The orexinergic neurons in the lateral hypothalamic area that promote arousal are particularly of interest in R6/2 mice as their population is reduced and their circadian activity is disrupted (Williams et al., 2011).

A disruption in the methamphetamine-sensitive circadian oscillator (MASCO), which relies primarily on the dopaminergic system (Blum et al., 2014), has been reported in HD mice (Cuesta et al., 2012; Ouk et al., 2016a). Since the MASCO is characterized by increases in period length, a MASCO deficit in the R6/2 mice might also underlie their inability to respond to changes in photoperiod that require lengthening their endogenous period.

Although some studies reported retinal alterations in HD patients (Paulus et al., 1993; Kersten et al., 2015), to our knowledge, none investigated a circadian behavior in parallel. Bright-light therapy has been shown to be efficient in delaying circadian disruption in R6/2 mice by improving day–night rhythms (Cuesta et al., 2014), even more so when it is coupled with restricted periods of voluntary exercise. Circadian disruption has already been associated with sleep disruption and reduced cognitive performance but also with disease states such as cancer, metabolic diseases, and neurocognitive and mood disorders (Sahar and Sassone-Corsi, 2009; Kyriacou and Hastings, 2010; Lyall et al., 2018). In HD patients, circadian desynchronization has been reported to worsen clinical symptoms such as irritability, depression, and cognitive impairment (Aziz et al., 2010; Diago et al., 2018).

Given that the current treatment for HD is limited to a pharmacological treatment for chorea, research on the efficacy of bright-light therapies in HD patients to improve disease management and quality of life is warranted, particularly since bright-light therapy has positive effects on mood and sleep in patients with Parkinson’s disease and Alzheimer’s disease (Dowling et al., 2007; Willis and Turner, 2007). This alternative and noninvasive approach therapy has considerable potential to help reduce the socioeconomic burden of such disease on our society.

In conclusion, we show that R6/2 mice exhibited specific behavioral impairment in response to phase-delaying paradigms such as photic stimuli during early night and entrainment to a 26 h T-cycle, while exhibiting apparently normal behavior in response to phase-advancing paradigms such as photic stimuli during early night and entrainment to a 22 h T-cycle. Our results show that although progressive retinal abnormalities are seen in R6/2 mice, they are able to respond appropriately to light, provided that it was given at an appropriate phase of the circadian timing system and that the intensity and duration of the photic information were sufficient. If photic entrainment abnormalities are part of the circadian abnormalities exhibited by HD patients, then bright-light therapies administered at strategically determined times of day carry a potential for improving circadian dysfunction and related comorbidities in such patients.

## Synthesis

Reviewing Editor: Cheryl Sisk, Michigan State University Decisions are customarily a result of the Reviewing Editor and the peer reviewers coming together and discussing their recommendations until a consensus is reached. When revisions are invited, a fact-based synthesis statement explaining their decision and outlining what is needed to prepare a revision will be listed below. The following reviewer(s) agreed to reveal their identity: Anthony Hannan.

This manuscript describes interesting new data on circadian abnormalities, and their regulation, in a transgenic mouse model of Huntington's Disease (HD). The results are complex in the sense that the response to phase-delaying stimuli was relatively impaired, yet there were no significant changes in response to phase-advancing pulses or non-standard T cycles. In addition, light-induced expression of cFos or Per1 appear to be normal in the R6/2 mutants. Reviewers agreed that the manuscript is generally well written and the data clearly presented. The results are certainly interesting for the circadian community, although not necessarily translatable in clinical terms because of the very specific experiments that have been performed. In addition, there was some concern that the overuse of circadian jargon might be inaccessible to the general readership. Therefore, please edit your manuscript to avoid technical terms so that non-specialist readers can more easily grasp the findings and to frame these findings in their potential clinical importance. Please also respond to the following comments and edit the manuscript accordingly.

1. The authors state that “Given the importance of circadian function to human behavior, such deficits have the potential to exacerbate symptoms of HD, particularly cognitive impairment and irritability.” The authors didn't show data of relevance to the cognitive impairment and irritability. Irritability is clinically linked to depression, one of the most common psychiatric symptoms in HD. Linking to these cognitive and psychiatric endophenotypes would strengthen the manuscript.

2. There is previous evidence regarding retinal changes in this model, and the circadian clock machinery in the SCN is disrupted at advanced stages of the modeled pathology, but the results presented here indicate that the interplay between these two mechanisms is quite complicated. The authors suggest that other brain areas might be involved here, but there is still no evidence for this claim.

3. In the introduction, the authors state that “Photic stimulation acutely induces the expression of Per genes in the SCN, demonstrating their involvement in light-induced circadian shifts”. Even if clock genes are necessary for this, light-induced expression alone does not demonstrate involvement in phase shifts; please temper this statement, e.g., “suggesting that per genes are involved...”, or cite empirical evidence for the statement.

4. The authors talk about “symptomatic” and “presymptomatic” mice, but this is not explained in the methods nor in the results.

5. It would be helpful to include a diagram to illustrate the experimental design.

6. Some of the experiments/groups appear underpowered to detect sex effects, despite the evidence of sexual dimorphism in mouse models of HD. The authors state that they didn't find differences between female and male mice. Are the authors confident of their conclusions in this regard?

7. In Figure 2, why were phase shifts shown for only the 14 weeks R6/2 mice and not earlier weeks?

8. Figure 3 needs error bars, and there should be separate data for the WT controls at each of the three time points.

9. For the in situ experiment, 3 WT female and 3 R6/2 female mice were used. This seems underpowered? Why no male mice? How was this small subset of each group selected? What was the negative control for the in situ? Exactly how did the authors measure the level of expression? The background signals in Figure 4 are variable - was there an adjustment/scaling for this? Micrographs from Fig. 4 are representative for this experiment. Can the authors provide some statistics here? Were pictures scanned and analyzed with ImageJ or any other package?

10. In Figure 5C, F and I, most of the error bars are not visible, presumably as they are hidden behind the data mean symbol. Can the authors reformat to make it clear to the reader that there are error bars for each datapoint, and so the variance can be visually assessed?

11. The authors claim that both WT and R6/2 mice entrained to the T22 cycle. However, Fig. 5E shows a very weak entrainment in the mutants, which is not sustained for the whole procedure (at least for this particular actogram) - somewhat similar to what they discuss regarding the T26 cycle.

12. Since there is a clear age-dependent effect on circadian rhythmicity in the mutants, it is very important to state that in the T cycle experiments, when the animals were effectively subjected to this paradigm (and even more when they were placed in DD), they were more than 9-weeks old. Even if this does not invalidate the data, how can we discriminate between an effect of visual sensitivity of the system and an aging-related mechanism? Similarly, the experiments in DD/LL (Fig. 8) might be influenced by a history-dependent effect (even if this is true for both genotypes). Again, by the end of the experiment the differences could be attributed to aging.

## References

[B1] Albrecht U (2006) Orchestration of gene expression and physiology by the circadian clock. J Physiol 100:243–251. 10.1016/j.jphysparis.2007.05.004 17643274

[B2] Albrecht U, Sun ZS, Eichele G, Lee CC (1997) A differential response of two putative mammalian circadian regulators, mper1 and mper2, to light. Cell 91:1055–1064. 10.1016/s0092-8674(00)80495-x 9428527

[B63] Aschoff J (1960) Exogenous and endogenous components on circadian rhythms. Cold Spring Harb Symp Quant Biol 25:11–28. 13684695 10.1101/sqb.1960.025.01.004

[B3] Aziz NA, Anguelova GV, Marinus J, Lammers GJ, Roos RAC (2010) Sleep and circadian rhythm alterations correlate with depression and cognitive impairment in Huntington’s disease. Park Relat Disord 16:345–350. 10.1016/j.parkreldis.2010.02.009 20236854

[B4] Bae K, Jin X, Maywood ES, Hastings MH, Reppert SM, Weaver DR (2001) Differential functions of mPer1, mPer2, and mPer3 in the SCN circadian clock. Neuron 30:525–536. 10.1016/s0896-6273(01)00302-6 11395012

[B5] Batcha AH, Greferath U, Jobling AI, Vessey KA, Ward MM, Nithianantharajah J, Hannan AJ, Kalloniatis M, Fletcher EL (2012) Retinal dysfunction, photoreceptor protein dysregulation and neuronal remodelling in the R6/1 mouse model of Huntington’s disease. Neurobiol Dis 45:887–896. 10.1016/j.nbd.2011.12.004 22198376

[B6] Bates GP, Dorsey R, Gusella JF, Hayden MR, Kay C, Leavitt BR, Nance M, Ross CA, Scahill RI, Wetzel R, Wild EJ, Tabrizi SJ (2015) Huntington disease. Nat Rev Dis Primers 1:15005. 10.1038/nrdp.2015.5 27188817

[B7] Blum ID, Zhu L, Moquin L, Kokoeva MV, Gratton A, Giros B, Storch K-F (2014) A highly tunable dopaminergic oscillator generates ultradian rhythms of behavioral arousal. Elife 3:e05105. 10.7554/eLife.05105 PMC4337656 25546305

[B8] Brown SA, Schibler U (1999) The ins and outs of circadian timekeeping. Curr Opin Genet Dev 9:588–594. 10.1016/s0959-437x(99)00009-x 10508692

[B9] Cermakian N, Monaco L, Pando MP, Dierich A, Sassone-Corsi P (2001) Altered behavioral rhythms and clock gene expression in mice with a targeted mutation in the Period1 gene. EMBO J 20:3967–3974. 10.1093/emboj/20.15.3967 11483500 PMC149149

[B10] Cheong RY, Gabery S, Petersén Å (2019) The role of hypothalamic pathology for non-motor features of Huntington’s disease. J Huntingtons Dis 8:375–391. 31594240 10.3233/JHD-190372 PMC6839491

[B11] Chou TC, Scammell TE, Gooley JJ, Gaus SE, Saper CB, Lu J (2003) Critical role of dorsomedial hypothalamic nucleus in a wide range of behavioral circadian rhythms. J Neurosci 23:10691–10702. 10.1523/JNEUROSCI.23-33-10691.2003 14627654 PMC6740926

[B12] Colwell CS, Foster RG (1992) Photic regulation of Fos-like immunoreactivity in the suprachiasmatic nucleus of the mouse. J Comp Neurol 324:135–142. 10.1002/cne.903240202 1430326

[B13] Cuesta M, Aungier J, Morton AJ (2012) The methamphetamine-sensitive circadian oscillator is dysfunctional in a transgenic mouse model of Huntington’s disease. Neurobiol Dis 45:145–155. 10.1016/j.nbd.2011.07.016 21820053

[B14] Cuesta M, Aungier J, Morton AJ (2014) Behavioral therapy reverses circadian deficits in a transgenic mouse model of Huntington’s disease. Neurobiol Dis 63:85–91. 10.1016/j.nbd.2013.11.008 24269914

[B15] Daan S, Pittendrigh CS (1976) A Functional analysis of circadian pacemakers in nocturnal rodents: II. The variability of phase response curves. J Comp Physiol 106:253–266. 10.1007/BF01417857

[B16] Decoursey PJ (1960) Phase control of activity in a rodent. Cold Spring Harb Symp Quant Biol 25:49–55. 10.1101/sqb.1960.025.01.006 13721130

[B17] Diago EB, Martínez-Horta S, Lasaosa SS, Alebesque AV, Pérez-Pérez J, Kulisevsky J, Del Val JL (2018) Circadian rhythm, cognition, and mood disorders in Huntington’s disease. J Huntingtons Dis 7:193–198. 10.3233/JHD-180291 29843249

[B18] Dowling GA, Graf CL, Hubbard EM, Luxenberg JS (2007) Light treatment for neuropsychiatric behaviors in Alzheimer’s disease. West J Nurs Res 29:961–975. 10.1177/0193945907303083 17596638 PMC2387134

[B19] Edelstein K, Amir S (1999) The role of the intergeniculate leaflet in entrainment of circadian rhythms to a skeleton photoperiod. J Neurosci 19:372–380. 10.1523/JNEUROSCI.19-01-00372.1999 9870966 PMC6782384

[B20] Gompf HS, Aston-Jones G (2008) Role of orexin input in the diurnal rhythm of locus coeruleus impulse activity. Brain Res 1224:43–52. 10.1016/j.brainres.2008.05.060 18614159 PMC2596878

[B21] Helmlinger D, Yvert G, Picaud S, Merienne K, Sahel J, Mandel J-L, Devys D, Helmlinger D, Mandel J-L, Devys D, Sahel J, Mandel J-L, Devys D (2002) Progressive retinal degeneration and dysfunction in R6 Huntington’s disease mice. Hum Mol Genet 11:3351–3359. 10.1093/hmg/11.26.3351 12471061

[B22] Johnson CH (1999) Forty years of PRCs–what have we learned? Chronobiol Int 16:711–743. 10.3109/07420529909016940 10584173

[B23] Kersten HM, Danesh-Meyer HV, Kilfoyle DH, Roxburgh RH (2015) Optical coherence tomography findings in Huntington’s disease: a potential biomarker of disease progression. J Neurol 262:2457–2465. 10.1007/s00415-015-7869-2 26233693

[B24] Ko CH, Takahashi JS (2006) Molecular components of the mammalian circadian clock. Hum Mol Genet 15:R271–R277. 10.1093/hmg/ddl207 16987893

[B25] Kornhauser JM, Mayo KE, Takahashi JS (1996) Light, immediate-early genes, and circadian rhythms. Behav Genet 26:221–240. 10.1007/bf02359382 8754249

[B26] Kudo T, Schroeder A, Loh DH, Kuljis D, Jordan MC, Roos KP, Colwell CS (2011) Dysfunctions in circadian behavior and physiology in mouse models of Huntington’s disease. Exp Neurol 228:80–90. 10.1016/j.expneurol.2010.12.011 21184755 PMC4346330

[B27] Kuhlman SJ, Silver R, Le Sauter J, Bult-Ito A, McMahon DG (2003) Phase resetting light pulses induce Per1 and persistent spike activity in a subpopulation of biological clock neurons. J Neurosci 23:1441–50. 10.1523/JNEUROSCI.23-04-01441.2003 12598633 PMC3281758

[B28] Kyriacou CP, Hastings MH (2010) Circadian clocks: genes, sleep, and cognition. Trends Cogn Sci 14:259–267. 10.1016/j.tics.2010.03.007 20418150

[B29] Li J-D, Hu W-P, Zhou Q-Y (2012) The circadian output signals from the suprachiasmatic nuclei. Prog Brain Res 199:119–127. 22877662 10.1016/B978-0-444-59427-3.00028-9

[B30] Lin M, Liao P, Chen H, Chang C, Chen S, Chern Y (2019) Degeneration of ipRGCs in mouse models of Huntington’s disease disrupts non-image forming behaviors prior to motor impairment. J Neurosci 39:1505–1524. 30587542 10.1523/JNEUROSCI.0571-18.2018 PMC6381252

[B31] Lyall LM, Wyse CA, Graham N, Ferguson A, Lyall DM, Cullen B, Celis Morales CA, Biello SM, Mackay D, Ward J, Strawbridge RJ, Gill JMR, Bailey MES, Pell JP, Smith DJ (2018) Association of disrupted circadian rhythmicity with mood disorders, subjective wellbeing, and cognitive function: a cross-sectional study of 91 105 participants from the UK Biobank. Lancet Psychiatry 5:507–514. 10.1016/S2215-0366(18)30139-1 29776774

[B32] Masana MI, Benloucif S, Dubocovich ML (1996) Light-induced c-fos mRNA expression in the suprachiasmatic nucleus and the retina of C3H/HeN mice. Brain Res Mol Brain Res 42:193–201. 10.1016/s0169-328x(96)00031-9 9013774

[B33] Maywood ES, Fraenkel E, Mcallister CJ, Wood N, Reddy AB, Hastings MH, Morton AJ (2010) Disruption of peripheral circadian timekeeping in a mouse model of Huntington’s disease and its restoration by temporally scheduled feeding. J Neurosci 30:10199–10204. 10.1523/JNEUROSCI.1694-10.2010 20668203 PMC6633357

[B34] Mieda M, Ono D, Hasegawa E, Okamoto H, Honma K, Honma S, Sakurai T (2015) Cellular clocks in AVP Neurons of the SCN are critical for interneuronal coupling regulating circadian behavior rhythm. Neuron 85:1103–1116. 10.1016/j.neuron.2015.02.005 25741730

[B35] Morin LP, Allen CN (2006) The circadian visual system, 2005. Brain Res Rev 51:1–60. 10.1016/j.brainresrev.2005.08.003 16337005

[B36] Morton AJ (2013) Circadian and sleep disorder in Huntington’s disease. Exp Neurol 243:34–44. 10.1016/j.expneurol.2012.10.014 23099415

[B37] Morton AJ, Wood NI, Hastings MH, Hurelbrink C, Barker RA, Maywood ES (2005) Disintegration of the sleep-wake cycle and circadian timing in Huntington’s disease. J Neurosci 25:157–163. 10.1523/JNEUROSCI.3842-04.2005 15634777 PMC6725210

[B38] Mrosovsky N (1999) Masking: history, definitions, and measurement. Chronobiol Int 16:415–429. 10.3109/07420529908998717 10442236

[B39] Nelson DE, Takahashi JS (1991) Sensitivity and integration in a visual pathway for circadian entrainment in the hamster (Mesocricetus auratus). J Physiol 439:115–145. 10.1113/jphysiol.1991.sp018660 1895235 PMC1180102

[B40] Ouk K, Aungier J, Morton AJ (2016a) Progressive gene dose-dependent disruption of the methamphetamine-sensitive circadian oscillator-driven rhythms in a knock-in mouse model of Huntington’s disease. Exp Neurol 286:69–82. 10.1016/j.expneurol.2016.09.007 27646506

[B41] Ouk K, Hughes S, Pothecary CA, Peirson SN, Morton AJ (2016b) Attenuated pupillary light responses and downregulation of opsin expression parallel decline in circadian disruption in two different mouse models of Huntington’s disease. Hum Mol Genet 25:5418–5432. 10.1093/hmg/ddw359 PMC5418835 28031289

[B42] Ouk K, Aungier J, Morton AJ (2017) Prolonged day length exposure improves circadian deficits and survival in a transgenic mouse model of Huntington’s disease. Neurobiol Sleep Circadian Rhythms 2:27–38. 31236493 10.1016/j.nbscr.2016.11.004 PMC6575567

[B43] Pallier PN, Maywood ES, Zheng Z, Chesham JE, Inyushkin AN, Dyball R, Hastings MH, Morton A (2007) Pharmacological imposition of sleep slows cognitive decline and reverses dysregulation of circadian gene expression in a transgenic mouse model of Huntington’s disease. Neurobiol Dis 27:7869–7878. 10.1523/JNEUROSCI.0649-07.2007 PMC6672877 17634381

[B44] Panda S, Sato TK, Castrucci AM, Rollag MD, DeGrip WJ, Hogenesch JB, Provencio I, Kay SA (2002) Melanopsin (Opn4) requirement for normal light-induced circadian phase shifting. Science 298:2213–2216. 10.1126/science.1076848 12481141

[B45] Paulus W, Schwarz G, Werner A, Lange H, Bayer A, Hofschuster M, Müller N, Zrenner E (1993) Impairment of retinal increment thresholds in Huntington’s disease. Ann Neurol 34:574–578. 10.1002/ana.410340411 8215245

[B46] Pendergast JS, Friday RC, Yamazaki S (2010) Photic entrainment of period mutant mice is predicted from their phase response curves. J Neurosci 30:12179–12184. 10.1523/JNEUROSCI.2607-10.2010 20826680 PMC2943870

[B47] Perez-Leon JA, Warren EJ, Allen CN, Robinson DW, Lane Brown R (2006) Synaptic inputs to retinal ganglion cells that set the circadian clock. Eur J Neurosci 24:1117–1123. 10.1111/j.1460-9568.2006.04999.x 16930437 PMC2435212

[B48] Petrasch-Parwez E, Habbes HW, Weickert S, Löbbecke-Schumacher M, Striedinger K, Wieczorek S, Dermietzel R, Epplen JT (2004) Fine-structural analysis and connexin expression in the retina of a transgenic model of Huntington’s disease. J Comp Neurol 479:181–197. 10.1002/cne.20327 15452853

[B49] Pittendrigh CS (1960) Circadian rhythms and the circadian organization of living systems. Cold Spring Harb Symp Quant Biol 25:159–184. 10.1101/sqb.1960.025.01.015 13736116

[B50] Pittendrigh CS, Daan S (1976) A functional analysis of circadian pacemakers in nocturnal rodents. IV. Entrainment: pacemaker as clock. J Comp Physiol 106:291–331. 10.1007/BF01417859

[B51] Ragauskas S, Leinonen H, Puranen J, Rönkkö S, Nymark S, Gurevicius K, Lipponen A, Kontkanen O, Puoliväli J, Tanila H, Kalesnykas G (2014) Early retinal function deficit without prominent morphological changes in the R6/2 mouse model of Huntington’s disease. PLoS One 9:e113317. 10.1371/journal.pone.0113317 25469887 PMC4254453

[B52] Ruby NF, Brennan TJ, Xie X, Cao V, Franken P, Heller HC, O’Hara BF (2002) Role of melanopsin in circadian responses to light. Science 298:2211–2213. 10.1126/science.1076701 12481140

[B53] Sahar S, Sassone-Corsi P (2009) Metabolism and cancer: the circadian clock connection. Nat Rev Cancer 9:886–896. 10.1038/nrc2747 19935677

[B54] Schobel SA, Palermo G, Auinger P, Long JD, Ma S, Khwaja OS, Trundell D, Cudkowicz M, Hersch S, Sampaio C, Dorsey ER, Leavitt BR, Kieburtz KD, Sevigny JJ, Langbehn DR, Tabrizi SJ (2017) Motor, cognitive, and functional declines contribute to a single progressive factor in early HD. Neurology 89:2495–2502. 10.1212/WNL.0000000000004743 29142089 PMC5729794

[B55] Schwartz WJ, Tavakoli-Nezhad M, Lambert CM, Weaver DR, de la Iglesia HO (2011) Distinct patterns of Period gene expression in the suprachiasmatic nucleus underlie circadian clock photoentrainment by advances or delays. Proc Natl Acad Sci U S A 108:17219–17224. 10.1073/pnas.1107848108 21969555 PMC3193200

[B56] Shearman LP, Zylka MJ, Weaver DR, Kolakowski LF, Reppert SM (1997) Two period homologs: circadian expression and photic regulation in the suprachiasmatic nuclei. Neuron 19:1261–1269. 10.1016/S0896-6273(00)80417-1 9427249

[B57] van Wamelen DJ, Roos RA, Aziz NA (2015) Therapeutic strategies for circadian rhythm and sleep disturbances in Huntington disease. Neurodegener Dis Manag 5:549–559. 10.2217/nmt.15.45 26621387

[B58] Vercruysse P, Vieau D, Blum D, Petersén Å, Dupuis L (2018) Hypothalamic alterations in neurodegenerative diseases and their relation to abnormal energy metabolism. Front Mol Neurosci 11:2. 10.3389/fnmol.2018.00002 29403354 PMC5780436

[B59] Williams RH, Morton AJ, Burdakov D (2011) Paradoxical function of orexin/hypocretin circuits in a mouse model of Huntington’s disease. Neurobiol Dis 42:438–445. 10.1016/j.nbd.2011.02.006 21324360 PMC5767114

[B60] Willis GL, Turner EJD (2007) Primary and secondary features of Parkinson’s disease improve with strategic exposure to bright light: a case series study. Chronobiol Int 24:521–537. 10.1080/07420520701420717 17612949

[B61] Wood NI, McAllister CJ, Cuesta M, Aungier J, Fraenkel E, Morton AJ (2013) Adaptation to experimental jet-lag in R6/2 mice despite circadian dysrhythmia. PLoS One 8:e55036. 10.1371/journal.pone.0055036 23390510 PMC3563662

[B62] Yan L, Silver R (2002) Differential induction and localization of mPer1 and mPer2 during advancing and delaying phase shifts. Eur J Neurosci 16:1531–1540. 10.1046/j.1460-9568.2002.02224.x 12405967 PMC3281755

